# Радикальное лечение диффузного токсического зоба у детей.

**DOI:** 10.14341/probl13086

**Published:** 2022-04-30

**Authors:** Т. Е. Иванникова, Т. Ю. Ширяева, Е. В. Нагаева, М. С. Шеремета, Д. Н. Бровин, О. Б. Безлепкина

**Affiliations:** Национальный медицинский исследовательский центр эндокринологии; Национальный медицинский исследовательский центр эндокринологии; Национальный медицинский исследовательский центр эндокринологии; Национальный медицинский исследовательский центр эндокринологии; Национальный медицинский исследовательский центр эндокринологии; Национальный медицинский исследовательский центр эндокринологии

**Keywords:** диффузный токсический зоб, гипертиреоз, тиреоидэктомия, радиойодтерапия

## Abstract

**ОБОСНОВАНИЕ:**

ОБОСНОВАНИЕ. Гипертиреоз, вызванный диффузным токсическим зобом (ДТЗ), является относительно редким заболеванием у детей. Существует два метода его лечения: консервативный, т.е. медикаментозная терапия, и радикальный: хирургическое лечение и радиойодтерапия (РЙТ). При неэффективности медикаментозной терапии рассматривается вопрос выбора радикального метода лечения: РЙТ или тотальной тиреоидэктомии.

**ЦЕЛЬ:**

ЦЕЛЬ. Оценка результатов радикального лечения детей с диффузным токсическим зобом.

**МАТЕРИАЛЫ И МЕТОДЫ:**

МАТЕРИАЛЫ И МЕТОДЫ. Ретроспективное и проспективное одноцентровое исследование, включающее 122 пациента с диффузным токсическим зобом, в том числе с узловыми образованиями на фоне ДТЗ, которым было проведено радикальное лечение (с 2016 г. по 2021 г.).

**РЕЗУЛЬТАТЫ:**

РЕЗУЛЬТАТЫ. Средний возраст на момент обследования составлял 13,5±3,5 года. Пациенты были разделы на 2 группы в зависимости от проведенного лечения: 1-ю группу (n=60) составили дети, которым проведено оперативное лечение, 2-ю группу (n=62) — дети, которым проведена РЙТ. Медиана дозы тиреостатических препаратов у детей обеих групп достоверно не различалась (р=0,06), дети, которым была проведена РЙТ, получали тиреостатическую терапию достоверно более длительное время (p=0,024). Эндокринная офтальмопатия (ЭОП) имелась у 58 детей (47,5%) и встречалась одинаково часто в обеих группах, однако активная стадия ЭОП имелась только у детей 1-й группы. У детей 1-й группы объем щитовидной железы был достоверно больше (р=0,004), только в 1-й группе имелись узловые образования щитовидной железы (p=0,0007).

**ЗАКЛЮЧЕНИЕ:**

ЗАКЛЮЧЕНИЕ. РЙТ можно считать эффективным и безопасным методом лечения ДТЗ. Эффективность РЙТ зависит в том числе от объема щитовидной железы, согласно результатам построенной ROC-кривой, риск повторного применений РЙТ выше при объеме более 55 см3. Также нежелательно проведение РЙТ при наличии офтальмопатии в связи с возможным ухудшением течения ЭОП. При оперативном лечении в раннем послеоперационном периоде в 20% случаев отмечался гипопаратиреоз, 5% — парез возвратного гортанного нерва. Пациентам с выявленными узловыми образованиями по результатам УЗИ предпочтительно проводить оперативное лечение в связи с невозможностью исключить рак щитовидной железы.

## Abstract (en)

**BACKGROUND:**

BACKGROUND: Hyperthyreoidism due to Graves’ disease is a rare disorder in pediatric practice. There is 2 treatment options in Graves’ disease: medical treatment and definitive treatment, including surgery and radioactive iodine. Each method has its advantages and disadvantages. If medical therapy is ineffective the choice between radical treatment method is raised: radioactive iodine or total thyroidectomy. In this research we analyze treatment outcomes in pediatric Graves’ disease patients after different radical treatment methods.

**AIM:**

AIM: Comparative analysis of radical treatment outcomes in pediatric patients with Graves’ disease.

**MATERIALS AND METHODS:**

MATERIALS AND METHODS: Retrospective and prospective one-center research of 122 patients with Graves’ disease after radical treatment (between 2016 and 2021)

**RESULTS:**

RESULTS: The mean age was 13.5±3,5 year at the moment of examination. Patients were divided into 2 groups due to the radical treatments method: 1 group (n=60) were children after surgical treatment, 2 group (n=62) — after radioactive iodine. The mean dose of medical treatment in these groups did not reliably differ (p=0,06), duration of the medical treatment was reliably longer in patients after radioactive iodine (p=0,024). Graves’ orbitopathy was diagnosed in 58 patients (47,5%) and met equally often in both groups, but active stage of Graves’ orbitopathy was diagnosed only in patients from the 1st group. Thyroid size was reliable bigger in patients from the 1st group (p=0,004), and thyroid gland nodes were diagnosed only in patients from 1st group (p=0,0007).

**CONCLUSION:**

CONCLUSION: RI can be considered an effective and safe treatment for GD. The effectiveness of RI depends on the volume of the thyroid gland; according to the results of the constructed ROC curve, the risk of repeated RI is higher with a volume of more than 55 cm3. Also radioactive iodine is undesirable if there is signs of ophatalmopathy due to its possible deterioration. According to the results of the study hypoparathyroidism after surgical treatment was diagnosed in 20%, recurrent laryngeal nerve injury was diagnosed after surgical treatment in 5% of patients. In patients with identified nodular goiter according to the results of ultrasound, surgical treatment is preferable due to the impossibility of excluding thyroid cancer.

## ВВЕДЕНИЕ

Частота встречаемости диффузного токсического зоба (ДТЗ) у детей составляет 5% всех случаев ДТЗ [1]. Общая заболеваемость у детей и подростков составляет около 4,58:100 000 в год, в возрасте до 15 лет заболеваемость ниже: 1–2,91:100 000 в год. ДТЗ встречается в 3,4 раза чаще у девочек, чем у мальчиков [2]. В возрасте до 5 лет распространенность примерно в 10 раз ниже, при этом соотношение девочек и мальчиков составляет 1,4. Это соотношение заметно увеличивается с возрастом, особенно на втором десятилетии жизни [2][3]. Заболеваемость ДТЗ у детей варьирует в разных странах, так, в Северной Европе уровень заболеваемости составляет 0,1 на 100 000 среди детей раннего возраста и 3 на 100 000 среди подростков [4], в Гонконге показатель составляет до 14:100 000, в США распространенность составляет 1:10 000 [5][6].

По данным формы федерального статистического наблюдения № 12 «Сведения о числе заболеваний, зарегистрированных у пациентов, проживающих в районе обслуживания медицинской организации», заболеваемость в РФ за 2018–2020 гг. стабильно составляет около 1,94:100 000 детского населения, ежегодно в нашей стране диагностируется 600–800 новых случаев.

Существует три основных метода лечения ДТЗ: консервативное лечение тиреостатическими препаратами, хирургическое лечение, терапия радиоактивным йодом (РЙТ). Каждый из методов лечения имеет свои преимущества и недостатки.

Медикаментозное лечение больных ДТЗ остается терапией 1-й линии в педиатрической эндокринологической практике. Консервативная терапия в первую очередь направлена на снижение избыточной продукции тиреоидных гормонов путем торможения их синтеза и секреции, что способствует устранению проявлений гипертиреоза и нормализации уровня гормонов. Основными препаратами, которые используют при терапии больных ДТЗ, являются карбимазол (его активный метаболит тиамазол) и пропилтиоурацил.

Длительность данной терапии, согласно отечественным клиническим рекомендациям, у детей должна составлять не менее 3 лет [7][8]. После 2 лет лечения ремиссия заболевания наблюдается в 20–30% случаев [9][10]. Вероятность ремиссии увеличивается при увеличении продолжительности консервативной терапии [9][11].

При неэффективности консервативной терапии встает вопрос о радикальном лечении.

Хирургическое лечение заболеваний щитовидной железы (ЩЖ) применяется более 150 лет. Н.И. Пирогов выполнил впервые резекцию перешейка ЩЖ в 1847 г. во время командировки на Кавказ [12]. Радиоактивный йод (смесь изотопов I130 и I131) впервые был применен S. Hertz пациентке с болезнью Грейвса в январе 1941 г., у детей РЙТ стала применяться с 1945 г. В госпитале Университета Калифорнии (Сан-Франциско, США) 18 пациентам в возрасте до 20 лет была проведена РЙТ, в том числе 5 детям — до 10 лет [13]. С 1982 г. активно начало функционировать специализированное отделение радиохирургического лечения открытыми радионуклидами в Институте медицинской радиологии АМН СССР (в настоящее время — Медицинский радиологический научный центр им. А.Ф. Цыба — филиал ФГБУ «НМИЦ радиологии» Минздрава России, г. Обнинск) для пациентов с раком ЩЖ. До 2010 г. это отделение было единственным на всю страну. В настоящее время в России имеются несколько подразделений, где проводится РЙТ (Москва, Архангельск, Красноярск, Челябинск, Тюмень и др.). C 2015 г. в ФГБУ «НМИЦ эндокринологии» Минздрава России начал функционировать отдел ядерной медицины, в котором проводится РЙТ при ДТЗ детям.

В настоящее время оперативное лечение рассматривается как метод лечения гипертиреоза у пациентов с большим объемом ЩЖ, документально подтвержденным или предполагаемым злокачественным новообразованием в ЩЖ и эндокринной офтальмопатией (ЭОП; от умеренной до тяжелой степени) [14].

Хирургическое лечение обеспечивает высокую эффективность и быстрое излечение от гипертиреоза, однако тотальная тиреоидэктомия сопряжена с определенными рисками, включая развитие гипопаратиреоза, возникновение дисфункции голосовых связок [15–17], и поэтому в ряде случаев уступает РЙТ в качестве радикального лечения ДТЗ [18–20].

РЙТ в педиатрической практике появилась относительно недавно и в настоящее время используется все более широко. Зарубежные исследования у детей и взрослых демонстрируют, что однократная РЙТ не всегда приводит к гипотиреозу и требуется повторное ее проведение [21–24].

В данном исследовании проанализированы результаты лечения детей с ДТЗ, которым были проведены различные методы радикального вмешательства.

## ЦЕЛЬ ИССЛЕДОВАНИЯ

Цель: сравнительная оценка результатов радикального лечения у детей с диффузным токсическим зобом.

## МАТЕРИАЛЫ И МЕТОДЫ

## Место и время проведения исследования

Место проведения. Обследование и лечение пациентов проводилось в Институте детской эндокринологии ФГБУ «НМИЦ эндокринологии» Минздрава России.

Время исследования. В исследование включены пациенты, находившиеся в детской клинике с января 2016 г. по сентябрь 2021 г.

## Изучаемые популяции (одна или несколько)

Критериями включения в исследование являлись: возраст от 0 до 17 лет, наличие ДТЗ, положительный титр антител к рецептору тиреотропного гормона (ТТГ) в дебюте заболевания.

Критерии исключения: наличие узлового токсического зоба.

## Дизайн исследования

Ретроспективное и проспективное одноцентровое исследование, включающее 122 пациента с ДТЗ, в том числе с узловыми образованиями на фоне ДТЗ, которым было проведено радикальное лечение (1-я группа — 60 пациентов, которым проведено оперативное лечение, и 2-я группа — 62 пациента, которым была проведена РЙТ).

## Описание медицинского вмешательства (для интервенционных исследований)

Всем пациентам было проведено комплексное клинико-гормональное и инструментальное обследование, включавшее: сбор анамнеза жизни и заболевания, ультразвукового исследование (УЗИ) ЩЖ, определение содержания в сыворотке крови ТТГ, свободного тироксина (СТ4), свободного трийодтиронина (СТ3) и антител к рецептору ТТГ (АТрТТГ).

## Методы регистрации исходов

УЗИ ЩЖ проводилось на аппарате Тoshiba Aplio 500 линейным датчиком PLT-1204ВХ с диапазоном частот: 7–18 МГц. Сканирование ЩЖ осуществлялось в В-режиме и с применением режима цветового допплеровского картирования (ЦДК). Производилось измерение трех размеров обеих долей ЩЖ (длина, ширина и переднезадний размер), объем ЩЖ вычислялся по формуле J. Brunn (1981 г.):

[ширина правой доли (см) × длина правой доли (см) × толщина правой доли (см) + ширина левой доли (см) × длина левой доли (см) × толщина левой доли (см)] × 0,479.

Оценивались структура ЩЖ, степень эхогенности, васкуляризация, наличие узловых образований.

Исследование гормонов в сыворотке крови проводилось в лаборатории гормонального анализа ФГБУ «НМИЦ эндокринологии» Минздрава России. Лабораторные исследования были выполнены на автоматическом иммунохемилюминесцентном анализаторе Architect i2000sr (Abbott).

С целью сравнения лабораторных данных, выполненных в ФГБУ «НМИЦ эндокринологии» Минздрава России и по месту жительства, проводился пересчет уровней ТТГ, СТ4 и СТ3, пмоль/л, с помощью калькулятора пересчета единиц измерения анализов: https://www.slimhauz.ru/stoimost/analizy/kalkulyator_analizov.

Сцинтиграфия ЩЖ проводилась в отделе радионуклидной диагностики и терапии, осуществлялась на гамма-камерах однофотонной эмиссионной компьютерной томографии (ОФЭКТ) Discovery NM630 и ОФЭКТКТ Discovery NM/CT670 ФГБУ «НМИЦ эндокринологии» Минздрава России с применением 99mTc-пертехнетата.

Необходимая для исследования доза радиофармпрепарата (РФП) рассчитывалась индивидуально с помощью калькулятора вводимой активности PedDose в МБк имКи (https://www.eanm.org/publications/dosage-calculator).

Сцинтиграфия проводилась через 15–20 мин после внутривенного введения РФП в положении пациента лежа на спине, детектор гамма-камеры располагался максимально близко над шеей. Время исследования 10 мин. Затем врачом-радиологом на рабочей станции Xeleris (GI) проводилась оценка функционального состояния ЩЖ визуально и с помощью рассчитываемого программой индекса захвата РФП ЩЖ.

Дозиметрическое планирование проводилось по назначению врача-радиолога на системе ОФЭКТ Discovery NM630 ФГБУ «НМИЦ эндокринологии» Минздрава России с введением трейсерной активности 131-йода, активностью от 5 до 10 МБк. Сцинтиграфия проводилась через 2 ч после введения РФП в режиме «все тело» и через 24 ч в режиме «статика». В рамках процедуры определялся индекс захвата 131-йода через 24 ч после введения трейсерной активности (%), уточнялся объем долей ЩЖ по сцинтиграфическим признакам по формуле:

0,163 × (0,785 × ширина правой доли (см) × длина правой доли (см))^(3/2),

рассчитывалась мощность поглощенной дозы в ЩЖ через 24 ч (Гр/ч) при введении планируемой терапевтической аблационной активности.

РЙТ проводилась в закрытом режиме «Активные палаты» отделения радионуклидной терапии. Терапевтическая активность назначается врачом-радиологом на основании дозиметрического планирования, анамнеза заболевания, исходных характеристик пациента (текущий гормональный профиль пациента, индекс захвата 131-йода, удельный индекс захвата 99mTc-пертехнетата, реакция на снижение дозировки тиреостатика, наличие ЭОП). Выписка пациента производилась по результатам измерения уровня мощности эквивалентной дозы на расстоянии 1 м от поверхности тела согласно нормам НРБ 99/2009.

## Сбор катамнестических данных

Разработаны анкеты для катамнестического наблюдения, которые были разосланы всем 122 пациентам. Ответы получены от 70 пациентов (57,4%).

## Этическая экспертиза

Проведение данного исследования одобрено локальным Этическим комитетом ФГБУ «НМИЦ эндокринологии» Минздрава России (протокол № 17 от 23.10.2019 г.).

## Статистическая обработка

Размер выборки предварительно не рассчитывался. Статистическая обработка материала проводилась с использованием программ Microsoft Office Excel 2010, PSPP и статистического пакета STATISTIСA (StatSoft, США). При нормальном распределении количественного признака данные представлены в виде среднего значения и стандартной ошибки среднего: M±SEM, если не указано другое. При отличном от нормального распределении количественного признака данные представлены в виде значения медианы и его интерквартильного размаха: Me (25–75 перцентили), если не указано другое.

Для сравнения 2 групп по количественным признакам рассчитывался критерий Стьюдента для параметрических выборок, для непараметрических — применялся тест Манна–Уитни. Взаимосвязь между двумя показателями оценивалась с использованием корреляционного анализа методом Спирмена. Для всех статистических методов значение р<0,05 считалось статистически значимым. Для оценки риска развития рецидива ДТЗ после проведения РЙТ использовался ROC-анализ с помощью программы PSPP.

## Нежелательные явления

В ходе исследования нежелательных явлений зафиксировано не было.

## РЕЗУЛЬТАТЫ

## Объекты (участники) исследования

В Институте детской эндокринологии с 2016 по 2021 гг. наблюдались 122 ребенка с ДТЗ после РЙТ и оперативного лечения. Возраст на момент обследования составил от 4,9 до 17,9 (13,5±3,5) года.

Оперативное лечение было проведено 60 пациентам (9 мальчиков и 51 девочка), средний возраст детей на момент манифестации заболевания составил — 10,3±3,7 года, возраст постановки диагноза — 10,4±3,7 года.

РЙТ была проведена 62 пациентам (7 мальчиков и 55 девочек), средний возраст детей на момент манифестации заболевания — 9,9±2,9 года, возраст постановки диагноза 10,2±2,9 года.

## Основные результаты исследования

Дебют диффузного токсического зоба у детей.

Самой частой жалобой на момент начала заболевания у детей была тахикардия (38,3%; n=46), в 30% случаев — жалобы на увеличение ЩЖ и психоэмоциональную лабильность (26,7%), у 20% пациентов — экзофтальм и снижение веса. Реже дети жаловались на тремор (15%), потливость (10%), частый жидкий стул (5%), ухудшение зрения (3,3%), выпадение волос (3,3%), повышение артериального давления (3,3%).

При гормональном обследовании по месту жительства медиана уровня ТТГ составила 0,01 [ 0,005; 0,03], медиана уровней СТ3 и СТ4 — 18,4 [ 11,9; 30,3] и 45,2 [ 34,2; 67,3]. У всех детей имелся повышенный титр АТрТТГ, медиана составила 16,1 [ 5,6; 26,0]. Медиана объема ЩЖ составляла 20,1 [ 12,8; 31,3].

Все дети были разделы на 2 группы в зависимости от проведенного впоследствии радикального лечения: 1-ю группу (n=60) составили дети, которым проведено оперативное лечение, 2-ю группу (n=62) составили дети, которым проведена РЙТ. В таблице 1 представлены данные лабораторно-инструментальных исследований у детей при первичной диагностике.

По данным, представленным в таблице 1, достоверных различий между 2 группами детей не отмечалось.

**Table:** Таблица 1. Лабораторно-инструментальные данные детей с ДТЗ в дебюте заболеванияTable 1. Laboratory and instrumental data of children with DTG at the onset of the disease

Показатель	Оперативное лечение	РЙТ	р
Кол-во детей, n	60	62	
Возраст, лет	10,4±3,7	10,2±2,9	0,55
ТТГ (0,43-4,2 мМЕ/л)	0,01 [ 0,005; 0,04]	0,01 [ 0,003; 0,02]	0,23
СТ4 (10,1-17,9 пмоль/л)	49,2 [ 37,0; 69,4]	44,7 [ 28,4; 56,5]	0,60
СТ3 (2,8-6,3 пмоль/л)	15,5 [ 10,0; 30,0]	25,5 [ 13,8; 38,0]	0,17
АТрТТГ (0-1,75 МЕ/л)	19,0 [ 8,0; 25,5]	12,2 [ 6,9; 36,1]	0,33
Объем ЩЖ, см3	21,7 [ 11,9; 32,9]	17,7 [ 13,0; 26,2]	0,10

Тиреостатическая терапия

При поступлении в ФГБУ «НМИЦ эндокринологии» Минздрава России все пациенты получали тиреостатическую терапию. Медиана срока лечения тиреостатиками в 1-й группе пациентов до проведения оперативного лечения составила 3,4 года [ 1,6; 4,6], медиана дозы тиамазола составляла 0,22 мг/кг/сут [ 0,13; 0,38]. Побочных явлений от тиреостатической терапии не было выявлено ни в одном случае.

Во 2-й группе медиана дозы тиамазола составляла 0,17 мг/кг/сут [ 0,13; 0,30], медиана срока лечения — 4,3 года [ 2,5; 5,6]. У 8 пациентов имелись побочные явления от проводимой тиреостатической терапии: у 5 детей — лейкопения и нейтропения, у 2 пациентов — аллергические реакции, у 1 ребенка — повышение уровня трансаминаз в крови.

Таким образом, длительность медикаментозной терапии до проведения радикального лечения была достоверно меньше у детей 1-й группы и составила 3,4 года против 4,3 года у детей 2-й группы, p=0,024. Медиана дозы тиреостатических препаратов у детей двух групп достоверно не различалась: 0,22 и 0,17 мг/кг/сут, р=0,06.

Эндокринная офтальмопатия у детей с ДТЗ

ЭОП среди 122 детей имелась у 58 (47,5% детей) и встречалась одинаково часто в обеих группах (табл. 2).

**Table:** Таблица 2. Данные детей с ДТЗ и эндокринной офтальмопатией, которым проведено радикальное лечениеTable 2. Data of children with DTG and endocrine ophthalmopathy who underwent radical treatment

Группа лечения	Наличие ЭОП	Стадия ЭОП	Возраст манифестации ДТЗ, лет	Возраст радикального лечения, лет	Объем ЩЖ, см3
активная	неактивная
Оперативное лечение	32	4	28	10,0 [ 7,3; 13,6]	14,6 [ 12,6; 16,7]	54,8 [ 29,2; 75,4]
РЙТ	26	0	26	9,9 [ 7,2; 12,1]	14,8 [ 12,4; 16,2]	29,2 [ 21,9; 47,8]
р	0,20	0,06	0,038	0,54	0,95	0,004

В 1-й группе ЭОП отмечалась у 32 детей, из них у 15 ДТЗ манифестировал в достаточно раннем возрасте (от 3 до 9 лет), у 17 пациентов — в возрасте старше 9 лет. До проведения оперативного лечения одному ребенку потребовалось лечение ЭОП: парабульбарно вводился бетаметазон.

Медиана возраста оперативного лечения пациентов с ЭОП 14,6 года [ 12,6; 16,7]. Высокая активность процесса ЭОП выявлена в 4 случаях (12,5%). Из них у 3 пациентов ДТЗ манифестировал в возрасте старше 10 лет, а у одного — в возрасте до 10 лет. У оставшихся 28 пациентов отмечалась неактивная стадия ЭОП.

Во 2-й группе ЭОП выявлена у 26 пациентов (41,9%), у всех отмечалась низкая активность процесса. У 13 пациентов ДТЗ манифестировал в возрасте 9–18 лет; у 13 пациентов — в возрасте 3–9 лет. Перед проведением РЙТ дополнительное лечение по поводу ЭОП не проводилось ни одному пациенту. В нашем исследовании ухудшение течения офтальмопатии, потребовавшее проведения пульс-терапии, отмечалось у 1 пациента после РЙТ (3,8%).

Таким образом, ЭОП с одинаковой частотой встречалась у детей в обеих группах, однако активная стадия ЭОП имелась только у детей 1-й группы. Получены достоверные различия в объеме ЩЖ, у детей 1-й группы объем ЩЖ был достоверно больше, р=0,004.

Возраст манифестации ДТЗ и возраст проведения радикального лечения достоверно не различались у детей двух групп (табл. 3)

**Table:** Таблица 3. Лабораторно-инструментальные данные детей с ДТЗ перед проведением радикального леченияTable 3. Laboratory and instrumental data of children with DTG before radical treatment

Показатель	Оперативное лечение	РЙТ	р
Кол-во детей, n	60	62	
Возраст, лет	13,9 [ 11,7; 16,7]	14,9 [ 12,5; 16,2]	0,32
ТТГ (0,43-4,2 мМЕ/л)	0,02 [ 0,002; 1,8]	0,22 [ 0,006; 2.1]	0,25
СТ4 (10,1-17,9 пмоль/л)	12,5 [ 9,3; 18,7]	12,0 [ 9,3; 15,0]	0,17
СТ3 (2,8-6,3 пмоль/л)	6,4 [ 5,3; 9,2]	5,3 [ 4,4; 7,2]	0,07
АТрТТГ (0-1,75 МЕ/л)	14,3 [ 6,2; 26,4]	6,2 [ 2,2; 16,8]	0,014
Объем ЩЖ, см3	55,0 [ 29,9; 73,5]	28,6 [ 22,0; 47,7]	0,016
Кол-во детей с узловыми образованиями в ЩЖ, n	10	0	0,0007
Кол-во детей с активной стадией ЭОП, n	4	0	0,03

Хирургическое лечение было проведено 60 детям (тотальная тиреоидэктомия в 96,7%; гемитиреоидэктомия в 3,3%). При выявлении пареза возвратного гортанного нерва с одной стороны во время оперативного лечения операция была выполнена в объеме гемитиреоидэктомии. Медиана возраста на момент оперативного лечения составила 13,9 года [ 11,7; 16,7]. У половины пациентов (51,7%; n=31) объем ЩЖ был более 50 см3, медиана объема ЩЖ составляла 55 см3 [ 29,9; 73,5]. При проведении предоперационного обследования у 16,7% пациентов выявлены узловые образования ЩЖ: в 10% (n=6) случаев выявлен одноузловой зоб, в 6,7% (n=4) — многоузловой зоб. По результатам морфологического исследования у 8 пациентов отмечался активно пролиферирующий коллоидный зоб, а у 2 пациентов с одноузловым зобом — папиллярная карцинома. РЙТ была проведена 62 пациентам. Медиана возраста на момент радикального лечения составила 14,9 года [ 12,5; 16,2]. У 11 пациентов (17,7%) отмечался объем ЩЖ более 50 см3, медиана объема ЩЖ составляла 28,6 см3 [ 22,0; 47,7]. Ни у кого узловых образований ЩЖ выявлено не было. Медиана активности йода 131 составила 920 мБк [ 720; 1100].

У части пациентов после тотальной тиреоидэктомии отмечались послеоперационные осложнения: у 12 пациентов развился гипопаратиреоз (у 3 — транзиторный, у 6 — постоянный), у 3 пациентов — парез возвратного нерва (у 2 — транзиторный).

Таким образом, медиана объема ЩЖ перед проведением радикального лечения была достоверно больше у детей 1-й группы, р=0,016, кроме того, отмечалась достоверная разница в уровне АТрТТГ между 2 группами пациентов (p<0,05). В 1-й группе было выявлено достоверно больше узловых образований ЩЖ по результатам предоперационного обследования (p=0,0007) и ЭОП в активной стадии (р=0,03).

Катамнестическое наблюдение после радикального лечения детей с диффузным токсическим зобом

Катамнестическое обследование было проведено 70 пациентам (40 после хирургического лечения и 30 после РЙТ).

Оперативное лечение проводилось под контролем нейромониторинга у 31 пациента. Из них в послеоперационном периоде парез возвратного нерва отмечался у 3 детей, у 2 из них голос восстановился в течение 2 мес после операции, судьба 1 ребенка неизвестна. Послеоперационный гипопаратиреоз развился у 12 детей (20%), катамнестические данные получены от 9 из них: у 3 пациентов гипопаратиреоз носил транзиторный характер (исчез через 6–12 мес после хирургического лечения), у 6 сохраняется и требует заместительной терапии.

Все пациенты после тиреоидэктомии находятся на терапии левотироксином натрия. Медиана дозы левотироксина натрия составила 125 мкг/сут [ 100; 150].

Во 2-й группе было проведено мониторирование данных у 30 детей. Гипотиреоз после проведения РЙТ развивался через 1–4 мес, у большинства детей через 1–2 мес, всем детям назначалась терапия левотироксином натрия. Медиана дозы левотироксина натрия составила 100 мкг/сут [ 75; 125]. Четырем пациентам потребовалось проведение повторной процедуры РЙТ, согласно результатам построенной ROC-кривой, риск повторного применений РЙТ выше при объеме более 55,05 см3. Во всех 4 случаях объем ЩЖ составлял более 55 см3 (67,8, 87,6, 94,5, 56 см3), после чего развился гипотиреоз (через 1–2 мес после повторной РЙТ). Медиана первой дозы РФП у данных пациентов составила 920 мБк [ 731,3; 1100]. Медиана активности РЙТ при повторном проведении — 575 мБк [ 512,5; 722,5].

## ОБСУЖДЕНИЕ

Тиреоидэктомия является эффективным радикальным методом лечения ДТЗ [25–30], однако во многих странах не самым часто используемым по сравнению с терапией радиоактивным йодом [31]. Несмотря на низкий процент рецидивов после тотальной тиреоидэктомии, пациенты с ДТЗ подвергаются повышенному риску послеоперационных осложнений, включая кровотечение, парез возвратного нерва и гипопаратиреоз [25][26].

Послеоперационный гипопаратиреоз — одно из тяжелых осложнений тиреоидэктомии [32][33]. В большинстве случаев гипокальциемия носит транзиторный характер, но повреждение паращитовидных желез может привести к стойкому гипопаратиреозу.

Транзиторная гипокальциемия чаще развивается у пациентов с гипертиреозом из-за повышенного метаболизма костной ткани (синдром «голодных костей») [33]. Риск транзиторной гипокальциемии может быть снижен у пациентов с ДТЗ за счет предоперационного приема препаратов кальция [33]. В двух крупных ретроспективных исследованиях (R. Bellantone, 2002; E. Efremidou, 2009) сообщалось, что после тиреоидэктомии при ДТЗ транзиторная гипокальциемия наблюдалась у 7,3% пациентов, а постоянная — у 0,3–3,4% [34][35]. В нашем исследовании в послеоперационном периоде гипопаратиреоз развился у 20% пациентов (12 человек), катамнез показал, что как минимум у 6 из них он носит постоянный характер, у 3 — транзиторный характер.

Наиболее опасным осложнением тиреоидэктомии является повреждение возвратных гортанных нервов, приводящее к парезу голосовых связок и возможному нарушению дыхания, требующему трахеостомии. По данным зарубежной литературы отмечается, что риск постоянного пареза возвратного гортанного нерва составляет от 0 до 0,4%, а транзиторный парез возвратного гортанного нерва встречается у 1,3% пациентов [25]. Среди наших прооперированных пациентов послеоперационный парез возвратного гортанного нерва развился у 3 пациентов из 60 (5%), у 2 из них он носил транзиторный характер, в 1 случае информации получить не удалось.

Распространенность рака ЩЖ у пациентов с гипертиреозом изучается на протяжении многих лет, но точная связь между этими заболеваниями не установлена. Было высказано предположение, что гипертиреоз является фактором защиты от развития злокачественных новообразований. По другим данным, это частично связано с низким уровнем ТТГ, вызывающим подавление роста ткани ЩЖ и тем самым предотвращающее развитие роста раковых клеток [31]. Наоборот, высокий уровень ТТГ в сыворотке рассматривался в качестве фактора риска по развитию рака ЩЖ, и эксперименты in vitro показали, что ТТГ обладает способностью стимулировать рост фолликулярных клеток ЩЖ [31]. Метаанализ данных 28 исследований показал, что имеется положительная связь между повышенным уровнем ТТГ и уровнем злокачественности узловых образований ЩЖ [36]. В то же время недавние исследования показали, что может существовать связь между низким уровнем ТТГ в сыворотке и злокачественными новообразованиями и даже предполагают повышенную вероятность агрессивного течения у этих пациентов [37–41]. Перекрестные исследования в Европе и США с 2014 по 2017 гг. показали, что уровни ТТГ в сыворотке крови, находящиеся ниже нормального диапазона, связаны с повышенным риском рака ЩЖ [42][43]. Кроме того, общенациональное когортное исследование, проведенное в Дании, выявило повышенный риск развития рака ЩЖ у пациентов с гипертиреозом. Сделано предположение, что имитирующие ТТГ эффекты, вызванные циркулирующими АТрТТГ при ДТЗ, ответственны за повышенный риск злокачественной трансформации [44]. В исследовании, проведенном в Исландии, в качестве объяснения связи между уровнем ТТГ и раком ЩЖ рассматривается генетическая предрасположенность, а также предполагается, что низкие уровни ТТГ могут приводить к недостаточной дифференцировке клеток ЩЖ и способствуют высокой предрасположенности к мутациям и злокачественной трансформации [45]. По данным нашего исследования, у 16,7% пациентов были выявлены узловые образования (у 6 пациентов — одноузловой зоб, у 4 пациентов — многоузловой зоб). По результатам морфологического исследования папиллярная карцинома подтверждена у 2 из 10 детей, у остальных 8 детей по данным гистологического исследования — активно пролиферирующий коллоидный зоб.

Преимуществами РЙТ являются относительно низкая стоимость, простота введения препарата и относительная безопасность [46]. Относительным противопоказанием к проведению РЙТ является наличие ЭОП. В многочисленных исследованиях изучалось влияние РЙТ на развитие или прогрессирование ЭОП, полученные данные неоднозначны. В исследовании, проведенном А. Kung et al. [47], развитие или прогрессирование офтальмопатии в течение 2 лет наблюдения отмечалось у 22,8% пациентов в группе РЙТ и у 24, % в группе получавших тиреостатическую терапию [48]. Однако в исследовании L. Bartalena et al. [48] сообщалось, что частота развития или прогрессирования офтальмопатии была значительно выше в группе РЙТ (15,3%) по сравнению с группой на терапии метимазолом (2,7%), p<0,001.

В нашем исследовании ухудшение течения офтальмопатии, потребовавшее проведения пульс-терапии, отмечалось только у 1 пациента после РЙТ (3,8%).

Целью радикального лечения ДТЗ является развитие гипотиреоза. По данным исследований, после проведения РЙТ у детей эффект достигается у 95% пациентов [19][49], в нашем исследовании РЙТ была эффективной у 93,6% пациентов, 4 пациентам для достижения эффекта потребовалось повторное проведение РЙТ. Согласно результатам, риск повторного проведения РЙТ выше при объеме ЩЖ более 55,05 см3.

## ЗАКЛЮЧЕНИЕ

Таким образом, по результатам анализа данных можно судить, что РЙТ — эффективный и безопасный метод лечения ДТЗ у детей. Полная ремиссия заболевания достигается в 93,6% после первой процедуры в течение 5 лет и наступает в течение 1–4 мес после РЙТ. Эффективность РЙТ зависит от объема ЩЖ, более эффективна процедура при объеме менее 55 см3. При большем объеме риск повторной процедуры возрастает.

Тотальная тиреоидэктомия при ДТЗ приводит к ликвидации симптомов сразу после оперативного лечения. После хирургического лечения в 75% случаев осложнений не наблюдается, в 20% развивается гипопаратиреоз (в 33,3% — транзиторный). Проведение операции под контролем нейромониторинга позволяет снизить риск развития пареза возвратного гортанного нерва, но не гарантирует отсутствие осложнения, которое в нашем случае наблюдалось в 5%. Наличие узловых образований на фоне ДТЗ не исключает рак ЩЖ.

## ДОПОЛНИТЕЛЬНАЯ ИНФОРМАЦИЯ

Источники финансирования. Работа выполнена по инициативе авторов без привлечения финансирования.

Конфликт интересов. Авторы декларируют отсутствие явных и потенциальных конфликтов интересов, связанных с публикацией настоящей статьи, о которых необходимо сообщить.

Участие авторов. Иванникова Т.Е., Безлепкина О.Б., Ширяева Т.Ю., Шеремета М.С., Бровин Д.Н. — концепция и дизайн исследования; Шеремета М.С. — проведение радиойодтерапии; Бровин Д.Н. — проведение хирургического лечения; Иванникова Т.Е. — написание текста, статистическая обработка данных; Безлепкина О.Б., Ширяева Т.Ю., Шеремета М.С., Бровин Д.Н. — редакция текста, внесение ценных замечаний. Все авторы одобрили финальную версию статьи перед публикацией, выразили согласие нести ответственность за все аспекты работы, подразумевающую надлежащее изучение и решение вопросов, связанных с точностью или добросовестностью любой части работы.
